# Target volume delineation of anal cancer based on magnetic resonance imaging or positron emission tomography

**DOI:** 10.1186/s13014-017-0883-z

**Published:** 2017-09-06

**Authors:** Espen Rusten, Bernt Louni Rekstad, Christine Undseth, Ghazwan Al-Haidari, Bettina Hanekamp, Eivor Hernes, Taran Paulsen Hellebust, Eirik Malinen, Marianne Grønlie Guren

**Affiliations:** 1 Department of Physics, University of Oslo, Oslo, Norway; 2 Department of Medical Physics, Oslo University Hospital, Oslo, Norway; 3 Department of Oncology, Oslo University Hospital, Oslo, Norway; 4 Department of Radiology and Nuclear Medicine, Oslo University Hospital, Oslo, Norway; 5 K.G. Jebsen Colorectal Cancer Research Centre, Oslo University Hospital, Oslo, Norway; 6 Department of Medical Physics, Box 4953 Nydalen, N-0424 Oslo, PO Norway

## Abstract

**Purpose:**

To compare target volume delineation of anal cancer using positron emission tomography (PET) and magnetic resonance imaging (MRI) with respect to inter-observer and inter-modality variability.

**Methods:**

Nineteen patients with anal cancer undergoing chemoradiotherapy were prospectively included. Planning computed tomography (CT) images were co-registered with 18F–fluorodexocyglucose (FDG) PET/CT images and T2 and diffusion weighted (DW) MR images. Three oncologists delineated the Gross Tumor Volume (GTV) according to national guidelines and the visible tumor tissue (GTV_T_). MRI and PET based delineations were evaluated by absolute volumes and Dice similarity coefficients.

**Results:**

The median volume of the GTVs was 27 and 31 cm^3^ for PET and MRI, respectively, while it was 6 and 11 cm^3^ for GTV_T_. Both GTV and GTV_T_ volumes were highly correlated between delineators (*r* = 0.90 and *r* = 0.96, respectively). The median Dice similarity coefficient was 0.75 when comparing the GTVs based on PET/CT (GTV_PET_) with the GTVs based on MRI and CT (GTV_MRI_). The median Dice coefficient was 0.56 when comparing the visible tumor volume evaluated by PET (GTV_T_PET_) with the same volume evaluated by MRI (GTV_T_MRI_). Margins of 1–2 mm in the axial plane and 7–8 mm in superoinferior direction were required for coverage of the individual observer’s GTVs.

**Conclusions:**

The rather good agreement between PET- and MRI-based GTVs indicates that either modality may be used for standard target delineation of anal cancer. However, larger deviations were found for GTV_T_, which may impact future tumor boost strategies.

## Background

Squamous cell carcinoma of the anal canal is a rare cancer where the primary treatment is combined radiotherapy (RT) and chemotherapy, with surgery being reserved for salvage treatment [1]. Five year survival rate for patients with an early stage disease is around 80–90%, colostomy-free survival of advanced stages (T3-T4) is 50–60%, and about 20% of the patients experience relapse. The main challenge is local control, though a general escalation of the RT treatment dose is associated with acute toxicity and late effects which potentially inhibit treatment and impact quality of life [2]. To reduce toxicities smaller fields and conformal dose delivery, through intensity modulated RT (IMRT), has been introduced [3, 4]. As a result there is an increased emphasis on correct and precise delineation of the target volume [5, 6].

The RT target volume originates from the gross tumor volume (GTV) and includes regions with possible subclinical disease together with margins accounting for patient motion and setup. RT volumes such as GTV are commonly defined on Computed Tomography (CT) images suitable for dose calculations. Tumor stage is evaluated by tumor diameter and degree of infiltration into normal tissue, often visualized by Magnetic Resonance Imaging (MRI) with its high resolution and soft-tissue contrast in pelvic tumors [7]. Positron Emission Tomography (PET) provides high sensitivity in detecting presence of tumor, regional nodal status and distant metastatic spread [8], though in general has somewhat lower resolution compared to CT and MRI. The American clinical practice guidelines, National Comprehensive Cancer Network (NCCN), recommend that PET/CT should be considered for RT planning [9]. Ideally CT, MRI and PET would be co-registered and used for target volume definition, with PET aiding tumor localization and MRI aiding tumor border delineation [10]. In practice, even though all modalities may be used during staging and description of tumor extent, the target volume delineator may be presented with PET-CT, MR-CT, or CT only.

To differentiate the dose levels between elective target volumes and gross disease for anal carcinomas simultaneous integrated boost with IMRT has been used with good results [4, 11]. A further step in personalized treatment is to treat the target volume as an inhomogeneous structure, with different dose requirements due to cellular density and treatment resistance, and shaping dose distributions accordingly - dose painting. In this context both PET and MRI are evaluated as markers for local treatment resistance and for defining dose painting targets [12, 13]. For both squamous cell carcinomas of the head and neck and non-small cell lung cancer, studies have shown that recurrence tends to originate within regions with a high [18F]fluorodeoxyglucose (FDG) uptake [14, 15] and dose painting trials with PET have been initiated for these tumor types. However, at the moment no conclusive evidence has been presented [16, 17].

In the current study, target volumes have been delineated for a cohort of patients with anal cancer according to either MRI/CT or PET/CT. This has been conducted to compare the existing inter observer variability of each modality with the differences between imaging modalities. The purpose of this analysis is to evaluate the effect of each of the imaging modalities for target delineations in current clinical practice and in the context of future dose painting.

## Methods

### Patients

This prospective study (NCT01937780) includes 19 consecutive patients that were treated for squamous cell carcinoma of the anal canal at Oslo University Hospital between December 2013 and November 2014. Written, informed consent was obtained from all patients and the study was approved by the regional ethical committee. The patients had a median age of 64 years (range 40–88), a mean weight of 65 kg (range 52–91), and 84% were female. Tumor stage was T2/T3/T4 in 53/21/21% of the cases, and 63% of the patients had node positive disease (Table 1). One T0 patient had large nodal metastases after surgery of carcinoma in situ which was treated as a primary tumor. IMRT/VMAT or 3-D conformal radiotherapy was delivered with 46 Gy to the clinical target volume (CTV) together with one or two cycles of concomitant Mitomycin C and 5-fluorouracil depending on disease stage [18]. For T1-2 N0 tumors the primary tumor was given 54 Gy, while for T3–4 or N+ disease the GTV and pathological lymph nodes were given 58Gy. Imaging was performed prior to RT and comprised contrast-enhanced planning CT, T2-weighted (T2 W) and diffusion weighted (DW) MRI, and FDG-PET.

**Table 1:** Patient and tumor characteristics

		#N	Percent
Sex	Female	16	84
	Male	3	16
TNM	T0^a^	1	5
	T2	10	53
	T3	4	21
	T4	4	21
	N0	8	42
	N1	1	5
	N2	8	42
	N3	2	11
Stage	II	7	37
	IIIa	2	10
	IIIb	10	53
		Median	Range
Age(Years)		64.1	[40 88]
HIV^b^		2	10

^a^One patient with removed carcinoma in situ in anal canal with a large mesorectal lymph node metastases treated as a primary tumor

^b^The HIV positive patients had a T2N0 disease

Anonymized images were exported to the Eclipse RT planning system (Varian medical systems) where they were used for RT target volume delineation. For each patient the CT images were duplicated creating two cases with corresponding information on clinical examination, anorectoscopy and multidisciplinary team notes. The time between planning CT and PET, and planning CT and MRI acquisition was in median 4 (range 1–26) and 8 (range 3–17) days, respectively. In addition one case included MR imaging reports for MR delineations and the other PET imaging reports for PET delineations. A random order list of these 37 anonymized cases was created to minimize patient recognition and intra observer bias.

### Imaging

Medical imaging was conducted according to existing clinical protocols. PET examinations were performed after 6 h of fasting, using a Siemens Biograph 16 PET/CT scanner (Siemens, Erlangen, Germany). Images were reconstructed with OSEM 2i21s with a 3D PSF, TOF and a Gaussian 2 filter. A 400 × 400 reconstruction matrix was used with 2 mm resolution and 3 mm slice spacing. Images were obtained 1 h (62 min [55–80]) after an injection of ^18^FDG (255 MBq [168–366]). For all patients the glucose level was acceptable (5.3 mmol/l [4.4–6.8]). MR imaging consisted of an axial T2-weighted turbo spin echo (TSE) sequence (flip angle 90, TR 3712 ms, TE 80 ms, one echo, acquisition matrix 480 × 470, reconstruction matrix 672 × 672, pixel size 0.36 mm, field of view 240 mm, slice thickness 5 mm, slice gap 6 mm) and DWI (flip angle 90, TR 6843 ms, TE 65 ms, one echo, acquisition matrix 160 × 270, reconstruction matrix 320 × 320, pixel size 1.25 mm, field of view 400 mm, slice thickness 4 mm, slice gap 4 mm, b values 0, 10, 20, 40, 80, 160, 200, 400, 800, 1000, 1200, 1500). Images were obtained from a Philips Ingenia 3.0 T MR (Philips, Amsterdam, Netherlands) without a contrast agent. CT images where obtained from a General Electric LightSpeed Pro 16 (GE Healthcare, Chicago, Illinois, USA) with an Iomeron contrast agent (135 ml), axial resolution <1 mm and slice spacing 2.5 mm.

### Contouring

**Fig. 1:**
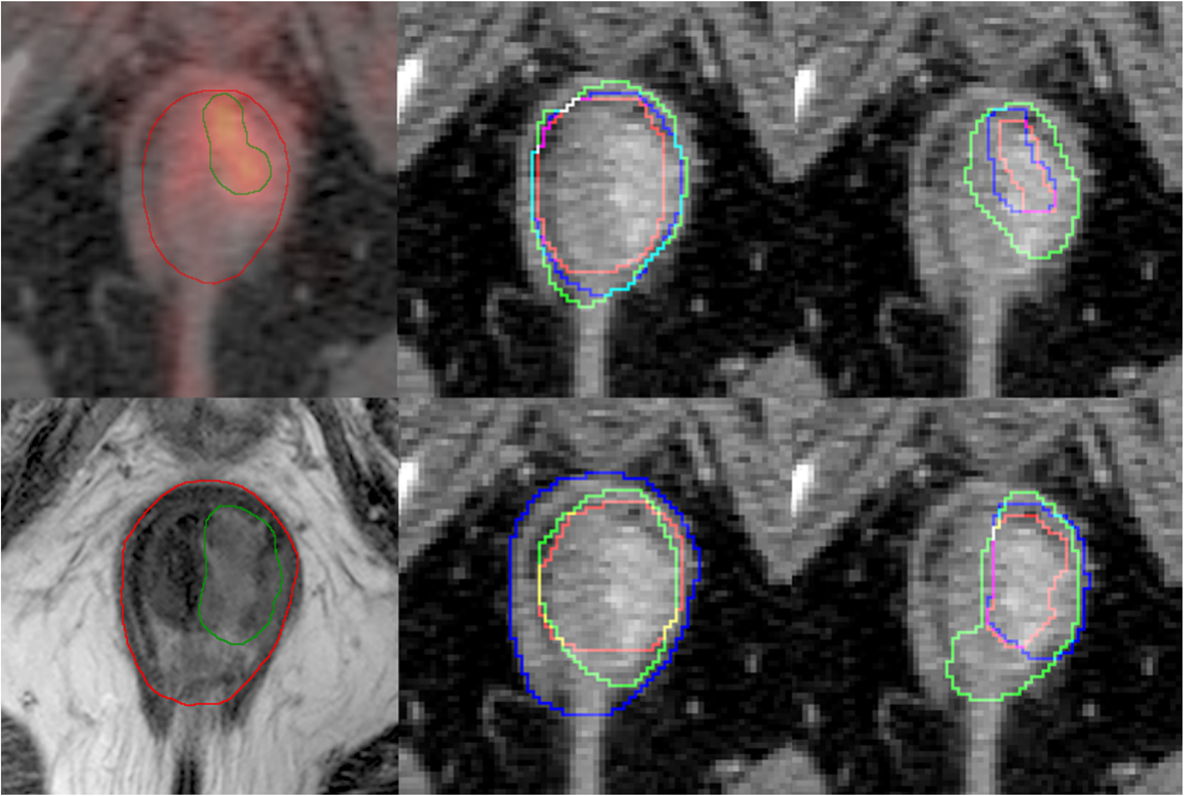
Delineations in one patient; all images from the same slice. Top row shows PET-based delineations. The left image is a single observer delineation of GTV (red) and GTV_T_ (green) in the PET/CT basis. The central image compares the three observers’ GTVs with each other, and the right compares the GTV_T_s. These two latter panels show delineations in the planning CT basis. The bottom row shows three corresponding MRI-based delineations

Three experienced oncologists (A, B, and C) independently performed the tumor delineations (Fig. 1). Delineation of lymph nodes was not considered in this work. All contours where defined on the CT image basis used for dose planning, while the auxiliary modality, MRI or PET, was shown as a blended/alternate image. The use of blended images or two modalities in two windows was left to the preference of the oncologist though the images were always relative to the CT. The co registrations were performed in Eclipse using a pelvic limited rigid mutual information registration. Notable shifts in anatomical structures were noted and the quality of the co registration was evaluated in terms of location of soft tissue structures in the anorectal region by each delineator on a scale 1–3 (1:poor, 2: acceptable, 3:good, decimals allowed). To limit the effect of the variable experience the delineators had in using the different modalities a fixed window was used for PET images during PET delineations and DWI was limited to b1500 values with a fixed window during MRI delineations. As such, the PET delineations reflected anatomical structures seen on the CT together with hypermetabolic regions seen on the PET. MRI delineations reflected anatomical structures seen on CT and T2 W-MRI together with hypo-diffusive regions seen on DWI. The degree of how the images were weighted was left to the clinical judgement of the oncologist. If necessary, the oncologists could discuss the tumor extent with a dedicated radiologist or nuclear medicine physician, as in accordance with routine clinical practice.

According to international practice on anal cancer [19] the GTV was delineated to include the visible tumor and the circumference of the anal canal and/or rectum when involved, as seen on axial T2 W/DW MR or PET images. Thus two GTVs were defined by each of the three observers for each patient; GTV_MRI_ and GTV_PET_. Furthermore, the visible macroscopic tumor (GTV_T_) on either MRI or PET was delineated as separate structures (GTV_T_MRI_/GTV_T_PET_)_._ Any shift in DW images relative to T2 W images, possibly due to the image sequence duration and geometric distortions, was adjusted for according to anatomical structures seen in the CT images.

### Analysis

DICOM images and structure sets were exported from the RT planning system and analyzed by in-house software developed in IDL (Exelis Visual Information Solutions). For each patient the total volume and center point of the GTVs and GTV_T_s were calculated. Three pairwise intersection volumes between doctors (A-B/B-C/C-A) and the three intersection volumes for each doctor between PET and MRI were calculated. The intersection volumes were normalized against the average volume of the two shapes, producing the Dice similarity coefficient [5]. The average Dice coefficient between the three doctor pairs represented the inter-observer variability for a given modality, while the average Dice coefficient for the three doctors between modalities represented the inter-modality variability. Furthermore, coronal, sagittal, and axial slices were reconstructed and a two dimensional Dice analysis was conducted, producing a profile over the mediolateral, anteroposterior and superoinferior directions. For each case the tumor width, as number of slices containing parts of the tumor, was normalized to one allowing an average curve over all patients to be produced. Margins, covering most of the observed variability, were calculated by expanding the median observer contour with an anisotropic kernel until it covered 90% of each individual observer contour.

For statistical analysis, Pearson’s correlations between volumes were calculated. Dice distributions were compared using the Wilcoxon Signed-rank test. Analysis of variance (ANOVA) was conducted to evaluate the influence of patients, doctors and imaging modalities. A *p* value less than 0.05 was considered statistically significant.

## Results

Delineations were completed on average in 20 min and the quality of the co-registration to the CT planning basis was deemed acceptable for both PET (2.5/3) and MRI (2.2/3). The most commonly reported issues were the position of the intestines, bladder filling and pelvic rotation. The median delineated GTV volumes of all patients was 27 (range 8–173) cm^3^ for PET and 31 (range 10–150) cm^3^ for MRI, with corresponding median GTV_T_ volume values of 6 (range 1–80) cm^3^ for PET and 11 (range 1–102) cm^3^ for MRI. Individual patient data are given in Table 2. For each patient the delineated PET and MRI volumes were highly correlated for both GTV (*r* = 0.95) and GTV_T_ (*r* = 0.94).

**Table 2:** Mean gross tumor volume (GTV) and active tumor region (GTV_T_) for three observers delineating target volumes using PET or MRI. Dice coefficients for inter observer PET, inter observer MRI, and intra observer comparing PET with MRI

			GTV					GTV_T_		
	Volume		Dice			Volume		Dice		
Pas	PET	MRI	PET	MRI	PET-MR	PET	MRI	PET	MRI	PET-MR
1	118	143	0.88	0.84	0.84	62	84	0.83	0.83	0.76
2	173	150	0.86	0.77	0.83	70	102	0.86	0.79	0.68
3	70	65	0.84	0.79	0.85	47	50	0.81	0.64	0.72
4	36	23	0.80	0.67	0.70	5	7	0.60	0.55	0.52
5	13	17	0.85	0.60	0.74	4	10	0.64	0.72	0.49
6	11	18	0.73	0.72	0.49	3	9	0.47	0.59	0.20
7	21	19	0.59	0.70	0.70	4	7	0.36	0.69	0.53
8	27	40	0.85	0.81	0.71	3	15	0.73	0.65	0.42
9	36	36	0.84	0.84	0.69	15	16	0.72	0.80	0.60
10	26	39	0.79	0.73	0.69	6	20	0.73	0.65	0.42
11	9	16	0.64	0.69	0.58	4	6	0.56	0.54	0.40
12	14	16	0.71	0.79	0.69	3	9	0.62	0.75	0.43
13	64	85	0.90	0.76	0.77	27	51	0.82	0.81	0.66
14	26	20	0.78	0.76	0.72	7	11	0.46	0.69	0.52
15	23	30	0.70	0.63	0.67	6	7	0.72	0.73	0.53
16	8	10	0.58	0.69	0.68	1	1	0.53	0.67	0.44
17	32	32	0.82	0.75	0.80	6	10	0.63	0.61	0.51
18	49	48	0.84	0.77	0.84	19	21	0.76	0.78	0.78
19	108	118	0.51	0.60	0.82	46	73	0.77	0.72	0.73

The impact of observer, imaging modality and individual patient on the absolute volumes was further investigated by ANOVA. Differences between patients in tumor volume was the primary feature in the ANOVA (*p* < 0.001), indicating that uncertainties in delineation were smaller than differences between patients for all delineators and modalities. The secondary feature was that delineated volumes were significantly larger for MRI compared to PET, both for GTV (F = 4.8, *p* = 0.032) and GTV_T_ (F = 9.4, *p* = 0.003), indicating that variance between modalities were greater than between doctors. The third feature was that some doctors produced larger delineations than others for the GTVs (F = 3.2, *p* = 0.047) while not for the GTV_T_s (F = 2.7, *p* = 0.068). The latter indicates that for GTV_T_ the intra delineator variance was comparable to the variance between delineators.

The main offset between center points of the individual delineations and the median volume was in the superoinferior direction with a median of 5 mm for the GTVs, 6 mm for GTV_T_PET_, and 7 mm for GTV_T_MRI_. The largest deviations of 2–4 cm originated from disagreement in the extent of whether to include the anorectal wall or not. The anisotropic margins required to ensure 90% coverage between delineation and the median volume were on average [0.9, 0.3, 5] mm for GTV_PET_, [1.5, 0.2, 7.8] mm for GTV_MRI_, [1.2, 0.9, 7.4] mm for GTV_T_PET_, and [2.7, 0.7, 10.2] mm for GTV_T_MRI_ in the mediolateral, anteroposterior and superoinferior directions.

**Fig. 2:**
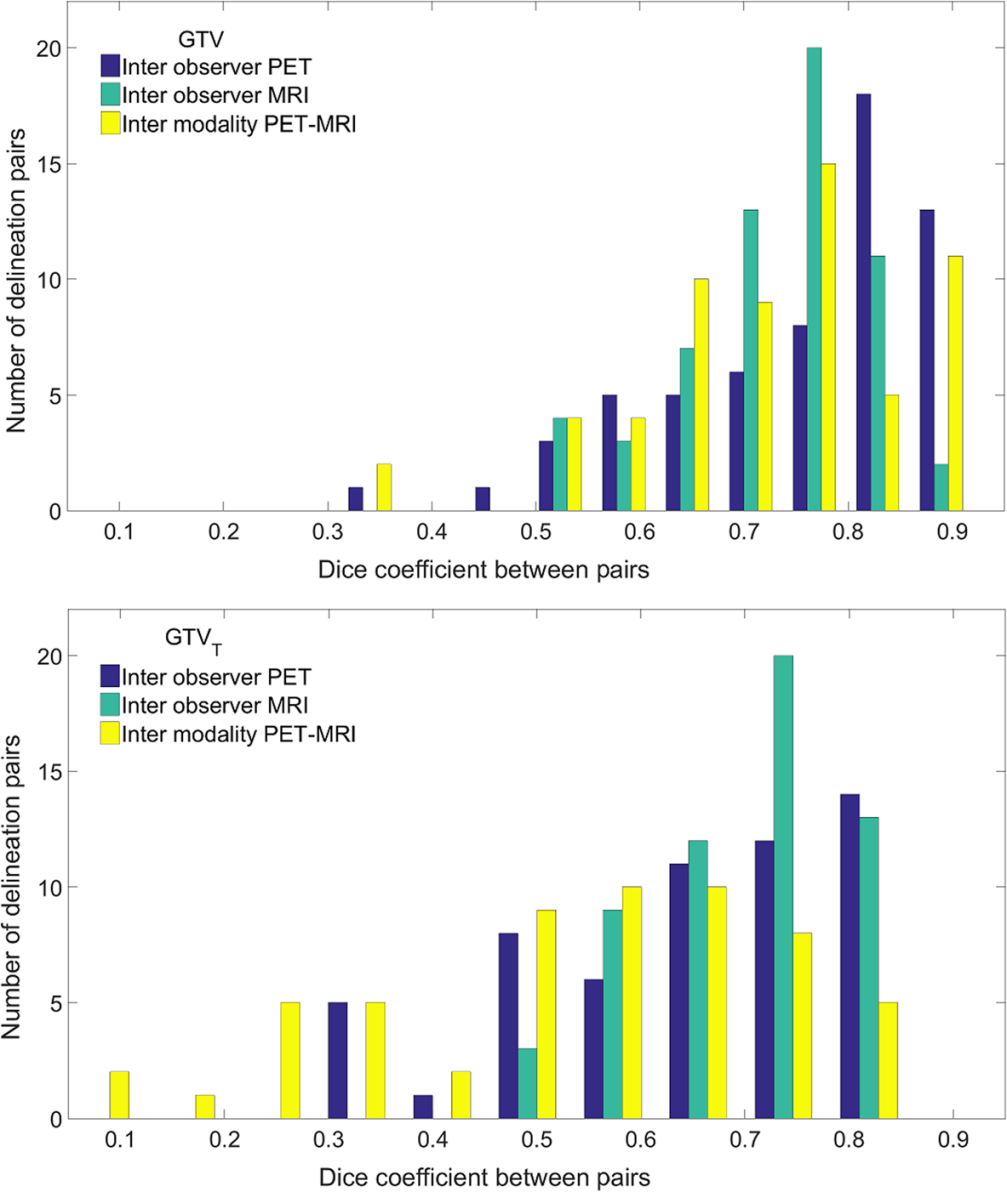
Dice similarity coefficient histograms for GTV (top) and GTV_T_ (bottom). The inter observer bars consist of 19 patients, each with three delineation pairs between doctors (AB, BC, CA). The inter modality bars are based on the PET delineations for each of the 19 patients paired with respective MR delineations of the same doctor

The distributions of Dice similarity coefficients over the current patient cohort, for inter-observer and inter-modality comparison, are presented in Fig. 2. The median Dice value of the three observer pairs was 0.80 (range 0.34–0.91) for GTV_PET_, 0.74 (range 0.53–0.86) for GTV_MRI_, and were not significantly different from each other (*p* = 0.053). For the PET-MRI inter-modality comparison a median Dice coefficient of 0.75 (range 0.31–0.92) was found. The inter-modality Dice distribution was significantly different from the PET inter-observer distribution (*p* = 0.04), but was not different from the MRI inter-observer distribution (*p* = 0.92). The inter-observer GTV_T_PET_ distribution, with a median value of 0.68 (range 0.3–0.86), was significantly different (*p* = 0.047) from GTV_T_MRI_ with a median of 0.71 (range 0.48–0.86), and both were significantly different (*p* < 0.001) from the PET-MRI inter-modality distribution with a median of 0.56 (range 0.04–0.81).

**Fig. 3:**
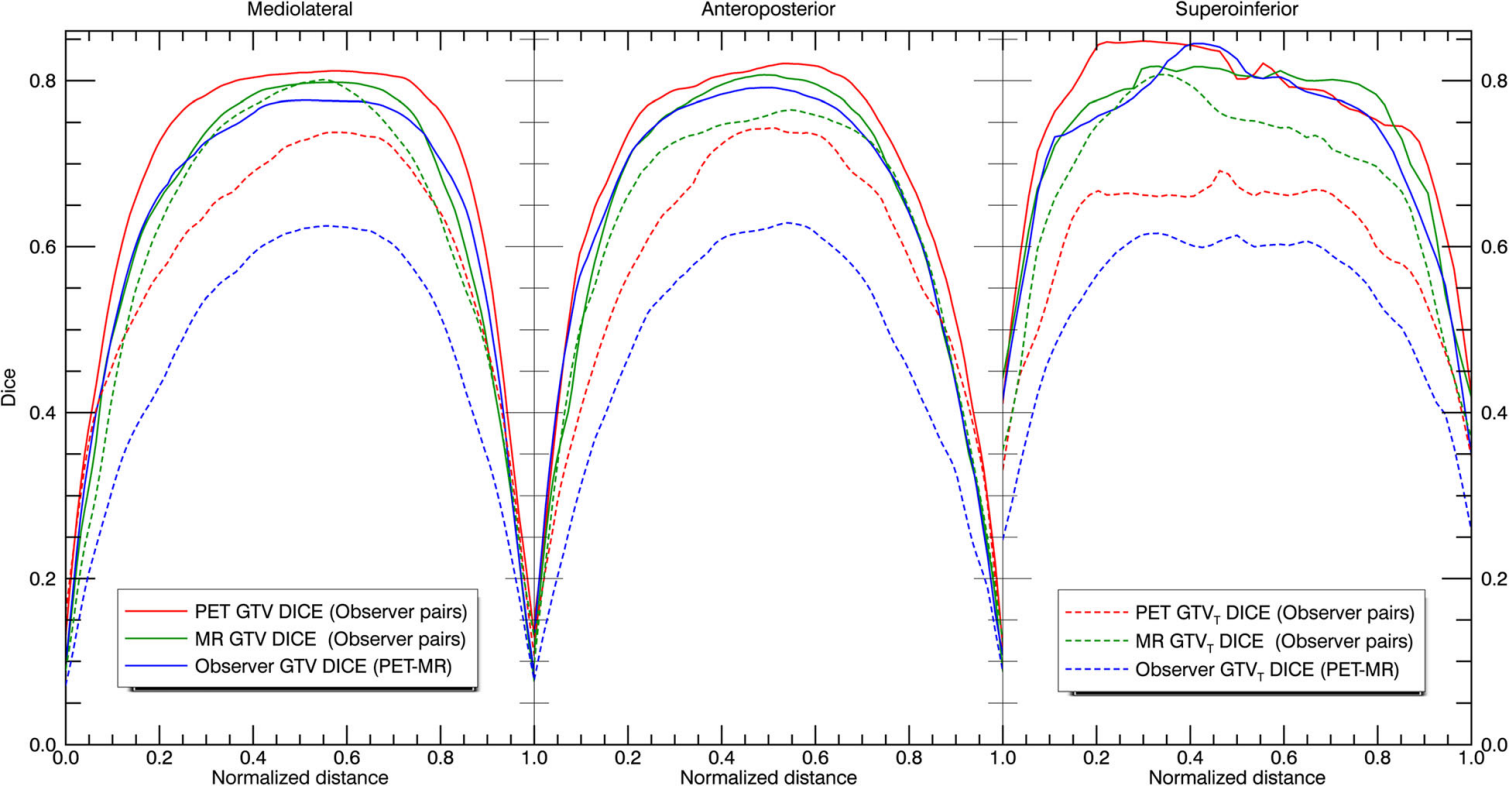
Profiles displaying the Dice similarity coefficient over the tumors in three orthogonal directions. All tumors have been normalized to unit width and Dice values are averaged over all patients

Figure 3 displays the Dice contours projected along the mediolateral, anteroposterior and superoinferior directions. In all directions the Dice values were lowest at the contour edges, and the shapes were relatively symmetrical. The superoinferior projection appears jagged as a result of the lower CT resolution in this direction. GTV_PET_ projections show higher values and appear broader than GTV_MRI_, while the opposite is true for the GTV_T_s. Inter-modality profiles are lower than the corresponding inter-observer profiles, and the difference is most pronounced for GTV_T_.

## Discussion

Generous margins have traditionally been applied in radiotherapy to correct for uncertainties in biology, movement, setup, delivery and imaging. Uncertainties in patient setup and delivery systems have steadily decreased over the years, while advances in medical imaging has been limited by co-registration issues and patient changes between sessions. The current work has focused on two main issues: how different observers will produce varying delineations for a given modality, and how their information from PET and MRI is interpreted differently.

MRI seemed to consistently produce slightly larger GTV volumes than PET, though the difference was small (median 4 cm^3^). Other studies have shown similar results, with MRI tending to overestimate GTV for rectal cancer, though clinical significance has yet to be established [20, 21]. The ANOVA and Dice distributions both demonstrated that the greatest uncertainty seemed to be the choice of modality and not the choice of observer. Although differences between delineators could be detected the Dice coefficients with a median of 0.8 for PET and 0.74 for MRI showed a high degree of overlap. The difference between delineators was seen as the same delineator repeatedly producing smaller, or larger, volumes than the others. This indicated that they were consistent in their own clinical judgement with low intra observer variability relative to inter observer or inter modality variability.

In the current work, GTV_T_ was delineated to encompass only the visible macroscopic tumor volume, as opposed to GTV which also included the anorectal wall. For PET/CT GTV_T_PET_ will largely be reflected by the hypermetabolic part of the tumor, as CT images provide sub-optimal images quality for this purpose. The hypermetabolic tumor area may be quantified in terms of the PET-based metabolic tumor volume (MTV), which has shown a prognostic role for anal cancer [22]. Since the GTV_T_PET_ includes MTV and is smaller than the GTV it is an attractive target for dose escalation due to smaller dosimetric impact on the surrounding normal tissue than a general escalation. GTV_T_MRI_ was contoured similarly with T2 W- and DW-MRI, thus including the most dense tumor tissue. For GTV_T_ the delineations seemed less consistent with respect to delineators and to how the modalities were interpreted. Dice values decreased to median values about 0.7 for both modalities, with a median inter modality Dice coefficient of 0.56. ANOVA could no longer distinguish delineators indicating that the doctors were less consistent in defining GTV_T_s compared to GTVs. Thus, local dose escalation based on either GTV_T_PET_ or GTV_T_MRI_ are expected to produce different dose distributions in the patient, but the clinical impact of these differences is not clear.

The differences in center points of the delineated volumes were 1–2 mm in the axial plane and 5–7 mm in the superoinferior direction, which was consistent with the margin required for 90% coverage of inter observer variability. The reason for the wide margin in the superoinferior direction was not only a result of disagreement on which slice to begin and end the tumor but also the lower out-of-plane resolution. No comparable study of anisotropic margins for anal cancer exists, though a recent study of lung cancer reported a required GTV-PTV margin of 3–6 mm to account for target delineation variability [23]. For rectum cancer the greatest margins are required in the anteroposterior direction, though this may be due to rectal motion and possibly the use of isotropic voxels [24]. In the current work, both differences in center of mass positions and margins were almost equal for GTV_PET_ and GTV_MRI_, with margins somewhat larger than the difference in center points. Both differences in center points and margins where greater for GTV_T_MRI_ than for GTV_T_PET_. This offset was somewhat counteracted by the larger MRI volumes producing greater overlap.

No previous inter-observer/modality delineation studies on anal cancer have been found in the literature, though it has been shown that PET is a useful supplement to CT [25–27]. For rectal cancer, inter-observer variability as measured by Dice coefficients has been shown to decrease when adding FDG-PET (0.81) to CT images (0.77) [28], while a comparison of the concordance index of CT + PET (0.82) and CT + MRI (0.79) did not report significant differences when switching between modalities [29]. These values are similar to our current results where the GTVs have a mean Dice coefficient of typically 0.75–0.8. Thus, the inter observer and inter modality variability in our work an anal cancer delineations seems comparable to that of rectal cancer.

## Conclusion

In summary, delineation based on PET or MRI produced similar GTVs for RT planning of anal cancer, though PET appears to have lower inter observer variability in terms of Dice coefficients. However, the deviations between PET and MRI-based delineations were not substantial and may not translate into clinically meaningful differences. Overall the GTV_T_s display greater variability than the GTVs, both between doctors and modalities. It appears that being presented both PET and MR images are not critical for current GTV delineations, although local dose escalation strategies (dose painting) targeting GTV_T_ may show greater dependence on imaging modality.

## Abbreviations

ANOVA: Analysis of variance
CT: Computed tomography
DW(I): Diffusion weighted (imaging)
F: Fisher statistic, the ratio between how far two distributions data are scattered from the mean
FDG: Fluorodeoxyglucose
GTV: Gross tumor volume
GTV_T_: Visible macroscopic tumor volume
IMRT: Intensity modulated radiotherapy
MRI: Magnetic resonance imaging
MTV: Metabolic tumor volume
N0–3: Tumor stage defining lymph node status
PET: Positron emission tomography
RT: Radiotherapy
T0–4: Tumor stage primarily reflecting tumor size
VMAT: Volumetric arc therapy
